# Lack of “obesity paradox” in patients presenting with ST-segment elevation myocardial infarction including cardiogenic shock: a multicenter German network registry analysis

**DOI:** 10.1186/s12872-015-0065-6

**Published:** 2015-07-11

**Authors:** Ibrahim Akin, Henrik Schneider, Christoph A. Nienaber, Werner Jung, Mike Lübke, Andreas Rillig, Uzair Ansari, Nina Wunderlich, Ralf Birkemeyer

**Affiliations:** Universitätsmedizin Mannheim, Mannheim, Germany; Universitätsklinikum Rostock und Hanseklinikum Wismar, .Rostock, .Germany; Universitätsklinikum Rostock, .Rostock, .Germany; Schwarzwald-Baar Klinikum Villingen-Schwenningen, .Villingen-Schwenningen, .Germany; Asklepios Klinikum St. Georg Hamburg, .Hamburg, Germany; Universitätsklinikum Rostock und Kardiovaskuläres Zentrum Darmstadt, Darmstadt, Germany; Universitätsklinikum Rostock und Herzklinik Ulm, Ulm, Germany; Medical Faculty Mannheim, University Heidelberg, Theodor-Kutzer Ufer 1-3, 68167 Mannheim, Germany

**Keywords:** Coronary stent, Obesity paradox, Mortality, Cardiogenic shock

## Abstract

**Background:**

Studies have associated obesity with better outcomes in comparison to non-obese patients after elective and emergency coronary revascularization. However, these findings might have been influenced by patient selection. Therefore we thought to look into the obesity paradox in a consecutive network STEMI population.

**Methods:**

The database of two German myocardial infarction network registries were combined and data from a total of 890 consecutive patients admitted and treated for acute STEMI including cardiogenic shock and cardiopulmonary resuscitation according to standardized protocols were analyzed. Patients were categorized in normal weight (≤24.9 kg/m^2^), overweight (25-30 kg/m^2^) and obese (>30 kg/m^2^) according to BMI.

**Results:**

Baseline clinical parameters revealed a higher comorbidity index for overweight and obese patients; 1-year follow-up comparison between varying groups revealed similar rates of all-cause death (9.1 % vs. 8.3 % vs. 6.2 %; p = 0.50), major adverse cardiac and cerebrovascular [MACCE (15.1 % vs. 13.4 % vs. 10.2 %; p = 0.53)] and target vessel revascularization in survivors [TVR (7.0 % vs. 5.0 % vs. 4.0 %; p = 0.47)] with normal weight when compared to overweight or obese patients. These results persisted after risk-adjustment for heterogeneous baseline characteristics of groups. An analysis of patients suffering from cardiogenic shock showed no impact of BMI on clinical endpoints.

**Conclusion:**

Our data from two network systems in Germany revealed no evidence of an “obesity paradox”in an all-comer STEMI population including patients with cardiogenic shock.

## Background

Obesity and associated disorders like hypertension, hyperlipidemia and diabetes are linked to increased morbidity and mortality among a Western population [1, 2]. This patient cohort is also at greater risk to develop coronary artery disease [2]. Population-based registry data revealed that 43 % and 24 % of coronary revascularizations were carried out in overweight and obese patients, respectively [3]. However, despite evidence of a positive correlation between obesity and increased cardiovascular morbidity, previous studies have described an “obesity paradox” in patients undergoing coronary revascularization either by interventional (PCI) or surgical (CABG) strategies, reporting a protective effect of obesity in terms of postoperative mortality. The first description of this phenomenon was done by Gruberg et al. 12 years ago [4]. Although the impact of obesity on clinical outcomes after elective PCI has been subsequently investigated in several studies, the issue remains controversial. Thus, there is insufficient data in unselected populations suffering from acute coronary syndrome (ACS), which is additionally associated with a complex thrombogenic and proinflammatory status [5–10]. Our current analysis compares clinical outcomes after PCI between consecutive normal weight, overweight and obese patients diagnosed with an ST-segment elevation myocardial infarction (STEMI) including patients with cardiogenic shock.

## Methods

### Network structures

Both myocardial infarction networks, which were the first networks in Germany, aim at coronary reperfusion therapy with primary PCI as the treatment prerogative for all presumed STEMI patients according to a uniform, regional treatment protocol patterned for a 24 h/7days week in a single interventional centre.

“Network A”, located in northeastern Germany constituted a mixed urban and rural catchment area with an approximate population of 415,000 inhabitants and was spread across a 60 km radius from its centre. At the time of collecting data, there were eight hospitals in the network area, with a lone high-volume interventional facility functioning as a 24 h/7days primary PCI service point. Emergency Medical Services (EMS) transferred suspected STEMI patients to the emergency department of the nearest hospital without prior announcement. Upon arrival of the patient, local emergency departments alarmed the interventional cardiology team and organized the direct transfer of the patient to the cathlab.

“Network B”, located in southwestern Germany, constituted a rural catchment area with approximately 350,000 inhabitants and was spread across a 35 km radius from its centre. At the time of data collection there were six hospitals in this network area, with a lone high-volume interventional facility functioning as a 24 h/7days primary PCI service point.

Trained personnel at all collection points supported both Network structures. All STEMI patients, irrespective of cardiogenic shock or preceding cardiopulmonary resuscitation were intended for primary PCI through femoral access.

### Primary PCI protocol

All provisionally diagnosed STEMI patients were treated with 250–500 mg Aspirin intravenously and received a weight adjusted unfractionated dose of Heparin (70 IU/kg) by EMS. The loading dose of clopidogrel (600 mg) was mostly administered before the PCI. In few cases, this was administered immediately after the procedure.

When treating patients in shock, interventional cardiologists were encouraged to treat all presumed hemodynamically relevant non-target lesions. Thrombectomy, periprocedural GPIIb/IIIa blockers (predominantly abciximab) and drug-eluting stents (DES) were utilized at the discretion of the operator. The full anticoagulant dosing of heparin was stopped after PCI, unless there was a high risk of thromboembolism (eg. atrial fibrillation or mechanical heart valves).

### Study population

Consecutive STEMI patients admitted for primary PCI were prospectively included in their respective registries, in network A from 2001 to 2003 and in network B from 2005 to 2007.

### Definitions

These were based on parameters defined by the World Health Organization (WHO) and the National Heart, Lung and Blood Institute. The patient population was classified into normal weight (body mass index [BMI] 18.5 - 24.9 kg/m^2^), overweight (BMI 25–30 kg/m^2^) and obese group (>30 kg/m^2^) [11, 12]. STEMI was diagnosed by the presence of chest pain lasting > 20 min and of significant ST-segment elevation (≥0.1 mV in two adjacent leads if leads I-III, aVF, aVL, V4-V6, and ≥ 0.2 mV in leads V1-V3), as in the first recorded electrocardiogram (ECG). Patients with persistent angina and presumably new left bundle branch block (LBBB) were included in the registry if myocardial infarction (MI) was subsequently confirmed. Cardiogenic shock was defined clinically by the presence of hypotension (systolic blood pressure < 90 mmHg for >30 min or need for vasopressors to maintain systolic blood pressure >90 mmHg) and tachycardia (heart rate >90 beats/min) with evidence of end-organ hypoperfusion [13]. Thrombolysis In Myocardial Infarction (TIMI) flow grades were assessed in the culprit vessel before and after the PCI procedure. Major bleeding was defined according to the TIMI major bleeding definition as intracerebral bleeding, bleeding requiring surgical intervention, bleeding requiring transfusion or loss of more than 5 g/% haemoglobin [14]. As indicators of guideline adherent therapy we analyzed pre- and in-hospital delays, procedural success of primary PCI, stent use, peri-interventional antiplatelet management, medication at discharge and medication at 12 months [15]. Procedural success was defined as residual stenosis < 30 % of the culprit lesion. For outcomes we analyzed mortality, re-infarction rate, target lesion revascularization (TLR) and target vessel revascularization (TVR) up to 12 months. TVR included repeat procedures, either PCI or CABG, in the target vessel. The composite of these events was defined as the major adverse cardiac and cerebrovascular events (MACCE) including death, MI, TVR and stroke. Patients were discouraged to undergo routine angiography for follow-up; therefore, all re-interventions can be counted as clinically driven. Stent thrombosis (ST) was classified according to the definition proposed by the Academic Research Consortium (ARC) [16].

### Data collection and follow-up

All patients diagnosed with STEMI were cataloged in a dedicated database. The procedure for follow-up usually included telephone interviews and subject-based questionnaires at the time frame of 6 and 12 months. A descriptive follow-up concerning mortality was obtained from state registries. The local ethics committees in Rostock (Medical Faculty of University Rostock, Germany) and Freiburg (Albert-Ludwigs-University Freiburg, Germany) approved the registries. All patients included in this study gave preemptive informed consent for the extension of our routine follow-up.

### Statistical methods

Data was analyzed according to established standards of descriptive statistics. Categorical variables were compared by chi^2^ test. Continuous variables were reported as mean ± standard deviation or median with interquartile ranges. For comparisons, the *t* test, the two-tailed Mann–Whitney *U* test and ANOVA model was used where appropriate. Odds ratios (OR) and 95 % confidence intervals (CI) were provided where appropriate. A *p* value of less than 0.05 was considered significant. A multivariate logistic regression analysis (stepwise backward model) including sex, age, diabetes, hypertension, smoking, renal failure, cardiogenic shock, resuscitation, stent type and impaired ejection fraction (<45 %) at discharge with normal weight as a fixed parameter was performed to determine independent factors predicting 12-month mortality and MACCE. The final logistic model for 12-month mortality with the independent variables age, diabetes and impaired ejection fraction (<45 %) at discharge showed a good predictive value (C-statistic = 0.84), and good calibration characteristics using the Hosmer-Lemeshow test (p = 0.90). Mortality and MACE at 12 months was adjusted for the above-mentioned variables. One-year survival was demonstrated by Kaplan-Meier curves and compared by log-rank test.

## Results

### Baseline characteristics and procedural outcomes

Our analysis is based on the 890 patients diagnosed with STEMI between 2001 and 2007 in this prospective study at the two participating centers. Patients were categorized as normal weight (n = 263), overweight (n = 432) and obese (n = 195) with a mean BMI of 23.2 ± 1.72 kg/m^2^ 27.2 ± 1.32 kg/m^2^, and 32.9 ± 2.83 kg/m^2^, respectively. Obese patients were younger than overweight and normal weight patients (65.7 ± 12.87 vs. 62.8 ± 11.70 vs. 60.4 ± 11.67; p < 0.0001) and had a higher comorbidity index with higher rates of diabetes (13.3 % vs. 19.5 % vs. 27.2 %; p < 0.006), and arterial hypertension (48.7 % vs. 63.9 % vs. 73.9 %; p < 0.0001), but with lower rates of impaired renal function (29.3 % vs. 18.7 % vs. 11.5 %; p < 0.0001) (Table 1).

**Table 1:** Baseline demographics of patients presenting with STEMI

	BMI			p Trend
	≤24.9	25-30	>30	
Patients (n)	263	432	195	<0.0001
Mean BMI, kg/m^2^ (SD)	23.2 (1.7)	27.2 (1.3)	32.9 (2.8)	0.11
Male (%)	73.0	79.9	75.9	
Age (SD), y	65.7 (12.8)	62.8 (11.7)	60.4 (11.7)	<0.006
Diabetes (%)				0.59
NIDDM	13.3	19.3	26.7	
IDDM	0	0.2	0.5	
Hypercholesterinemia (%)	45.2	45.8	49.7	<0.0001
Renal insufficiency (%)	29.3	18.7	11.5	<0.0001
Hypertension (%)	48.7	63.9	73.9	<0.0001
Smoking (%)				
Current	42.9	37.5	46.7	0.48
Previous myocardial infarction (%)	11.0	9.5	7.7	0.72
Previous PCI (%)	9.1	7.4	7.7	0.90
Previous CABG (%)	1.5	1.9	2.1	0.92
Previous stroke (%)	4.6	3.9	4.1	
Ejection fraction				0.88
>55 % (%)	32.5	30.7	36.1	
45-55 % (%)	33.5	37.9	36.2	
30-44 % (%)	28.4	25.8	22.0	
<30 % (%)	5.6	5.6	5.7	

**Legend**

*BMI* Body mass index, *CAD* Coronary artery disease, *PCI* Percutaneous coronary intervention, *NIDDM* Non-insulin dependent diabetes mellitus, *IDDM* Insulin-dependent diabetes mellitus, *CABG* Coronary artery bypass grafting

Data pertaining to pre-hospital and intra-hospital time intervals were also not different between groups. However we observed that normal weight and overweight patients suffered more often from cardiogenic shock (9.9 % vs. 11.1 % vs. 5.1 %; p = 0.02) and had a higher calculated Grace Score (100.6 ± 16.18 vs. 87.2 ± 12.56 vs. 74.2 ± 10.14; p = 0.02) (Table 2).

**Table 2:** Descriptive morphology of coronary artery disease in patients stratified according to body weight

	BMI			p Trend
	≤24.9	25-30	>30	
Vessel Disease (%)				
Single	47.1	48.0	50.5	0.78
Dual	28.8	28.4	29.4	
Triple	22.6	21.5	19.6	
Left main stenosis (%)	2.5	3.0	1.0	
Target vessel (%)				
LAD	48.1	44.0	47.2	0.55
LCX	16.5	16.7	11.4	
RCA	34.6	37.4	40.4	
LMCA	0.8	1.9	0.5	
Bypass graft	0	0	0.5	
TIMI-flow (%)				
0	62.0	55.8	65.6	0.65
1	8.2	8.4	8.3	
2	12.7	16.8	13.5	
3	17.1	19.0	12.6	
Grace score (%)	100.6 (87.6)	87.2 (77.6)	74.2 (53.8)	0.02
CPR (%)	9.1	8.1	5.1	0.25
Cardiogenic shock (%)	9.9	11.1	5.1	0.02
Fibrinolysis prior PCI (%)	1.1	1.2	2.6	0.35
Pain-to-door time, min (SD)	212.2 (164.0)	212.5 (154.6)	213.6 (165.2)	0.99
Door-to-lab time, min (SD)	30.2 (67.9)	27.9 (46.5)	26.0 (46.3)	0.73
Lab-to-balloon time, min (SD)	30.8 (15.21)	31.4 (16.5)	32.6 (15.6)	0.67

**Legend**

*LAD* Left anterior descending coronary artery, *LCX* Left circumflex coronary artery, *RCA* Right coronary artery, *LMCA* Left main coronary artery, *TIMI* Thrombolysis in myocardial infarction, *CPR* cardiopulmonary resuscitation

Approximately half of all patients included in this study had a multivessel coronary artery disease with no significant difference in the distribution of dual-vessel, triple-vessel and left-main vessel disease as well as treated target vessel (Table 2). Primary PCI, being performed through femoral access in all patients, with implantation of nearly 1.4 ± 0.9 stents per patient was carried out as a single vessel PCI in more than 90 % of cases without any change in strategy between groups. Use of DES was predominant in the study population. Normal weight patients presented more often with smaller vessel diameter (Table 3). Although anatomical and procedural characteristics including periprocedural complications were comparable the use of GP IIb/IIIa was more frequent in obese patients (81.3 % versus 85.7 % versus 94.3 %; p = 0.01).

**Table 3:** Procedural characteristics of patients receiving coronary intervention

	BMI			p Trend
	≤24.9	25-30	>30	
Primary PCI performed				
Single vessel PCI (%)	90.8	89.7	96.9	0.30
Multivessel PCI (%)	4.6	5.9	1.0	
Staged PCI (%)	23.2	21.8	24.7	
Stent details				
Number of implanted stents (SD)	1.4 (0.9)	1.4 (0.9)	1.5 (1.0)	0.82
Drug-eluting stents (%)	63.8	61.3	62.9	0.61
Diameter (mm)	3.0 (2.75-3.00)	3.0 (2.80-3.00)	3.0 (2.80-3.50)	0.030
Length (mm)	24.0 (20.0-35.0)	24.0 (20.0-38.0)	24.0 (22.0-36.0)	0.47
Postprocedural TIMI III (%)	84.1	82.3	82.3	0.29
Periprocedural complication (%)				
No-reflow	0	2.0	3.0	0.12
Complete AV-block	3.0	2.0	0	0.18
CPR	1.0	3.0	2.0	0.66
Stroke / TIA	1.0	0	0	0.74
Death	0	0	1.0	0.46
No complication during incex PCI	94.0	95.0	94.0	0.94
GP IIb/IIIA antagonist (%)	81.3	85.7	94.3	0.01

**Legends**

*TIMI* Thrombolysis in myocardial infarction, *GP* Glycoprotein, *PCI* percutaneous coronary intervention, *AV* atrioventricular node, *CPR* cardiopulmonary resuscitation, *TIA* transient cerebral ischemic attack

### In-hospital follow-up

The overall in hospital mortality rate was 5.3 % in the normal weight, 4.4 % in the overweight, and 3.1 % in obese groups (p = 0.51). Similarly, rates of MI, stroke and bleeding complications as well as need for repeat urgent revascularization and resuscitation was low with no differences between subsets (Table **4**).

**Table 4:** In-hospital and 1-year clinical follow-up of patients receiving stent implantation

	In-hospital follow-up			
	BMI			p Trend
	≤24.9	25-30	>30	
Death (%)	5.3	4.4	3.1	0.51
Myocardial infarction (%)	0.6	0	2.0	0.09
Stroke (%)	1.0	0	0	0.45
Repeat urgent revascularization (%)				
PCI	1.0	0	2.0	0.31
CABG	1.0	0	0	0.35
CPR (%)	2.0	2.0	0	0.41
Complete AV-block (%)	1.0	0	1.0	0.25
Aneurysma spurium (%)	1.0	2.0	0	0.43
Bleeding (%)				
Major	1.9	3.4	1.0	0.37
Minor	6.9	7.3	3.1	0.33
Insignificant	17.6	14.2	13.3	0.56
Triple antiplatelet therapy (%)	14.5	9.4	10.6	0.27
**One-year follow-up**				
Death (%)	9.1	8.3	6.2	0.50
Myocardial infarction (%)	5.7	4.7	5.1	0.92
Stroke (%)	0	1.3	1.0	0.37
MACCE (%)	15.1	13.4	10.2	0.53
TVR (%)	7.0	5.0	4.0	0.47
Definite ST according ARC (%)	4.0	2.0	1.0	0.26
ASS	70.6	69.0	74.4	0.66
Clopidogrel	46.9	37.6	48.9	0.13
Oral anticoagulation (%)	4.2	8.5	5.6	0.24

**Legends**

*PCI* Percutaneous coronary intervention, *CABG* Coronary bypass graft, *CPR* Cardiopulmonary resuscitation, *MACCE* Major adverse cardiac and cerebrovascular event, *TVR* Target vessel revascularization, *ST* Stent thrombosis, *ARC* Academic Research Consortium

### One-year follow-up

**Fig. 1:**
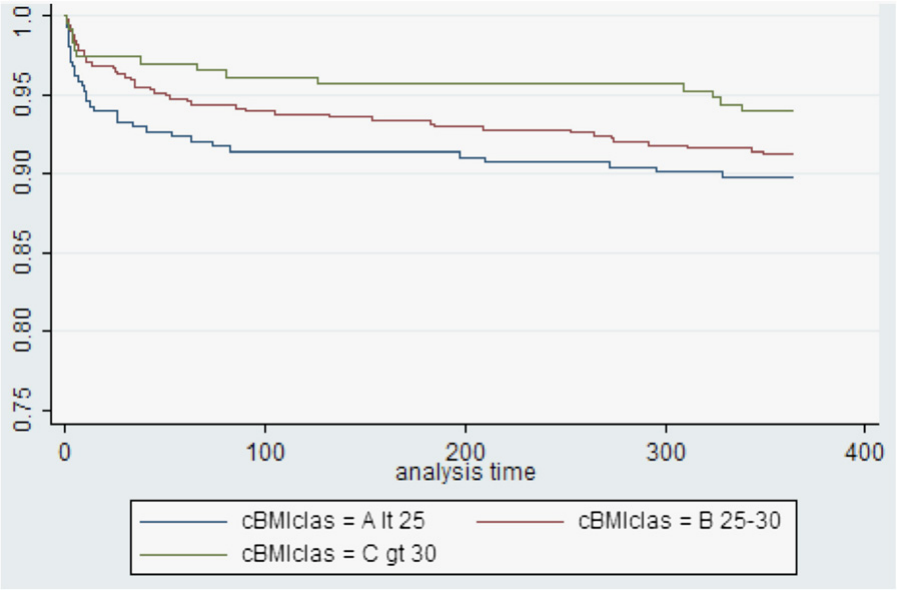
One-year overall survival of population stratified in normal weight, overweight and obese groups

At one-year follow-up no significant differences were noted between groups with respect to the incidence of MACCE-free survival and TVR-free survival. Similarly, no differences were noted in the rates of overall death, MI, stroke, and definite ST (Table 4, Fig. 1). The use of antiplatelet and anticoagulation treatment was not different between groups.

Subsequent risk-adjustment and multivariate analysis revealed no impact of overweight and obesity on clinical events. Predictors for one-year mortality were the presence of diabetes (OR 2.79; 95 % CI 1.50-5.19), age (OR 1.10; 95 % CI 1.06-1.14) and impaired left ventricular ejection fraction defined as < 45 % (OR 3.82; 95 % CI 2.05-7.13) without any impact of increasing BMI (OR 1.03; 95 % CI 0.62-1.42). Similarly, predictors for MACCE at follow-up were increasing age (OR 1.03; 95 % CI 1.01-1.06), while the use of a DES had protective effects (OR 0.44; 95 % CI 0.24-0.82) without impact of BMI (OR 0.91; 95 % CI 0.63-1.31).

An exclusion of lean patients (BMI < 18.5 kg/m^2^; n = 5) from the normal weight group did not change above-mentioned intrahospital and follow-up results with similar event rates in all three groups.

Additionally, a separate analysis of patients suffering from cardiogenic shock (26 versus 48 versus 8) did not show any differences between groups with a mean intrahospital and one-year follow-up mortality rate of up to 26 % and 31 %, respectively.

## Discussion

In Europe, the prevalence of obesity ranges from 4.0 to 36.5 % [17] and it is also well known that obesity acts as an independent cardiovascular risk factor for the development of coronary artery disease as well as general atherosclerosis and is associated with increased overall morbidity and mortality [18, 19]. There is evidence that this increased risk is mediated through obesity-related co-morbidities such as diabetes mellitus, hyperlipidemia, hypertension, increased insulin resistance, enhanced free fatty acid turnover, and promotion of systemic inflammation [20]. However, despite this correlation there is an assumption of an inverse correlation of obesity with mortality post PCI and less pronounced with a smaller need for repeat revascularisation. This has been described as the “obesity paradox” [10, 21]. An analysis of 9,633 patients being stratified in normal weight (n = 1,923), overweight (n = 4,813) and obese (n = 2,897) undergoing PCI revealed a higher incidence of major in-hospital complications, including cardiac death (1.0 % vs. 0.7 % vs. 0.4 %; p = 0.001) in normal weight than overweight and obese patients despite similar periprocedural data. This was driven by overall mortality (10.6 % vs. 5.7 % vs. 4.9 %; p < 0.0001). Cardiac mortality (4.8 % versus 3.3 % versus 2.5 %; p < 0.0001) was also significantly higher in normal weight patients; whereas rates of MI and TVR were similar [10]. A large meta-analysis including 250,152 patients with established coronary artery disease and a mean follow-up of 3.8 years supported these findings with increased relative risk for overall mortality [RR 1.37 (95 % CI 1.32-1.43)], and cardiovascular mortality [RR 1.45 (1.16-1.81)] after revascularization in normal weight patients [22]. These results persisted even after adjustment for potential confounders, including age, arterial hypertension, diabetes, and left ventricular function. Another analysis on patients with established coronary artery disease undergoing medical, interventional or surgical treatment showed an “obesity paradox” after revascularisation irrespective of the chosen strategy. In the whole cohort patients who were overweight or obese were more likely to undergo revascularization procedures compared with those with normal BMI, despite having lower risk coronary anatomy [23]. The underlying mechanism of the “obesity paradox” is speculative. Obesity is associated with lower levels of plasma renin, epinephrine and high serum levels of low-density lipoproteins that bind circulating lipopolysaccharides [24]. Coronary vessel diameters, as confirmed in out-patient cohorts, have been shown to correlate with the increase in body weight; thus a smaller coronary artery size in normal weight and lean patients could theoretically influence periprocedural outcome [25]. The relationship between obesity and survival is characterized in the literature by a J- or U-shaped curve with increasing mortality in the very lean or severely obese group [26, 27]; however, after adjustment for smoking and concurrent illness, the relationship has always been linear [28, 29]. Contrasting with these findings our analysis of high-risk all-comers STEMI population including patients with cardiogenic shock does not support the presence of an “obesity paradox”. Although there is a trend for better one-year survival in obese patients, this difference did not reach statistical significance. However, with access site being femoral there might be more bleeding events in obese patients, which could be avoided by radial access. Nevertheless, we think that the term “obesity paradox” might predominantly reflect different degrees of bias that cannot be completely corrected for by statistical means. Inherent bias in all obesity analyses result from the fact that overweight and obese patients are usually younger and have larger culprit coronary vessel diameters than normal weight counterparts. In general younger patients have better clinical outcomes after acute MI regardless of reperfusion modality [30, 31]. Additionally, the presence of co-morbidities in obese and overweight younger patients usually leads to more aggressive therapy of cardiovascular risk factors likely to improve outcomes despite obesity [30, 31]. In a study of 130,139 patients hospitalized for coronary artery disease, higher BMI was associated with increased use of standard medical therapies such as aspirin, beta-blockers, renin-angiotensin inhibitors, and lipid lowering therapy, and an increased likelihood of undergoing diagnostic catheterization and revascularization [32, 33]. The all-comer design of our registry with the majority of patients having had no established coronary artery disease before the index STEMI reduces the influence of potential confounders. Especially promotion of primary PCI in shock patients and after resuscitation (significantly more frequent in obese and overweight patients) avoided a severe pre-selection bias. Another point of discussion with respect to the obesity paradox is that underweight patients may receive standard anti-coagulation doses that are too high for their body size, making them more prone to post-procedural bleeding complications, which could be ruled out in our cohort by weight-adjusted doses [3, 5]. In addition obesity was found to correlate with higher levels of factor VII, VIII, fibrinogen and plasminogen activator inhibitor-1, which were all associated with increased risk of thrombosis [34]. Accordingly prospective investigations have shown that overweight and obese patients were more likely to suffer from suboptimal platelet response to clopidogrel and aspirin treatment [35, 36]. In our cohort the use of GP IIb/IIIa inhibitor was high facing the nature of exclusively high-risk STEMI patients. Furthermore obesity as well as STEMI is considered a low-grade inflammatory state, as demonstrated by increased levels of the pro-inflammatory cytokines interleukin-6 and tumor necrosis factor-alpha, and acute phase proteins such as C-reactive protein [37]. This proinflammatory state may also directly and indirectly cause thrombosis by oxidative stress and endothelial dysfunction [38]. Such findings could not be confirmed in our real-world setting with similar rates of stent thrombosis in all subsets. Since low BMI may be a marker of severe systemic illness [18, 39], we defined in a separate analysis the normal-weight group from 18.5 kg/m^2^-24.9 kg/m^2^ and excluded 5 extremely underweight patients. However, this did not change the previous findings with lack of an “obesity paradox”. A separate analysis of patients with cardiogenic shock, which is associated with a prothrombic situation and systemic inflammation, also revealed no statistical differences in clinical endpoints for all three groups.

### Study limitation

The present study is an observational non-randomized study in which patients were stratified according to their BMI at index-PCI. Thus, we had no information on intended or unintended weight change, as well as on variables like physical inactivity and socioeconomic factors which may have influenced the results. BMI is not as well correlated to cardiovascular disease and death as waist circumference and waist-to-hip ratio, which, however, were unavailable in our registries. Another limitation of our analysis is the length of follow-up and small sample size that might result in lack of power for meaningful conclusions but is reliable enough for hypothesis generation. An extended follow-up may result in a cumulative detrimental effect of obesity and may even manifest as increased late mortality and confirm the negative correlation of obesity with clinical outcomes even in a setting of coronary revascularization. Additionally the access site during PCI was femoral. With use of radial access site, bleeding events might be reduced in overweight and obese patients, which might result in better clinical outcomes as bleeding events correlate with overall mortality and myocardial infarction rate.

## Conclusions

Data from our all-comer network registry does not confirm the evidence of the “obesity paradox” during short and long term follow-up in patients suffering from STEMI including patients with cardiogenic shock. With respect to the limitations of available data prospective large-scale studies with long-term follow-up focusing on more reliable parameters reflecting the body fat are needed to reveal the phenomenon of the obesity paradox.

## Abbreviations

ACS: Acute coronary syndrome
ARC: Academic Research Consortium
BMI: Body mass index
CABG: Coronary artery bypass surgery
CI: Confidence interval
DES: Drug eluting stent
ECG: Electrocardiogram
EMS: Emergency medical service
LBBB: Left bundle branch block
MACCE: Major adverse cardiac and cerebrovascular events
MI: Myocardial infarction
OR: Odds ratio
PCI: Percutaneous coronary intervention
ST: Stent thrombosis
STEMI: ST elevation myocardial infarction
TIMI: Thrombolysis In Myocardial Infarction
TLR: Target lesion revascularization
TVR: Target vessel revascularization
WHO: World Health Organisation
